# Dabigatran accumulation in acute kidney injury: is more better than less to prevent bleeding? A case report

**DOI:** 10.1186/s12245-024-00677-3

**Published:** 2024-07-17

**Authors:** Rafik Matbouli, Olivier Pantet, Julien Castioni, Nima Vakilzadeh, Lorenzo Alberio, Olivier Hugli

**Affiliations:** 1 Emergency Department, Lausanne University Hospital & Lausanne University, BH 09-777/Bugnon 46, Lausanne, 1011 Switzerland; 2 Department of Adult Intensive Care, Lausanne University Hospital, Lausanne, Switzerland; 3 Department of Internal Medicine, Lausanne University Hospital, Lausanne, Switzerland; 4 Service of Nephrology and Hypertension, Department of Medicine, Lausanne University Hospital and University of Lausanne, Lausanne, Switzerland; 5 Service and Central Laboratory of Hematology, Department of Laboratory Medicine and Pathology, Lausanne University Hospital and Lausanne University, Lausanne, Switzerland

## Abstract

Dabigatran is an oral anticoagulant that is mainly renally excreted. Despite its efficacy in preventing thromboembolic events, concerns arise regarding bleeding complications in patients with acute kidney injury. Idarucizumab is its specific antidote and reverses quickly and effectively dabigatran anticoagulation effects in situations of severe bleeding or pending surgical procedures, but its benefit beyond these two indications remains uncertain. We present a case of a woman with atrial fibrillation anticoagulated by dabigatran and admitted with *Streptococcus agalactiae* meningitis, acute kidney injury and dabigatran accumulation. Idarucizumab was not administered initially as she did not meet its current strict indications. However, subsequently, significant bleeding necessitated its use. A rebound increase in dabigatran concentration was associated with an intracranial hemorrhage, but the combination of additional doses of idarucizumab with hemodialysis lowered the dabigatran concentration and prevented significant rebound increases. Further investigation into the optimal management of dabigatran accumulation and acute kidney injury-associated bleeding is needed to enhance patient outcomes and safety. Early initiation of hemodialysis together with idarucizumab administration may be crucial in preventing life-threatening bleeding events in these patients.

**Supplementary Information:**

The online version contains supplementary material available at 10.1186/s12245-024-00677-3.

## Introduction

Dabigatran is a direct thrombin inhibitor indicated for the treatment and prevention of arterial or venous thromboembolic events. It is used in around 6% of patients anticoagulated by a direct oral anticoagulant (DOAC) and accounts for 13.5% of total adverse events secondary to DOAC prescription in Europe. [1] Between 2016 and 2020, emergency department DOAC-related bleeding visits increased by 27.9%, amounting to 301,433 ED visits in 2020, with an estimated 6% due to dabigatran. [2] Its administration is contraindicated in the case of severe renal impairment as 80–85% is excreted via glomerular filtration. [3] Patients who develop acute kidney injury (AKI) are at increased bleeding risk due to dabigatran accumulation, but its anticoagulant activity can be quickly and rapidly reversed with its specific antidote, idarucizumab. Currently, idarucizumab is indicated for anticoagulated patients who have severe or uncontrollable bleeding events or require emergency surgical or non-surgical interventions with a high or unacceptable bleeding risk. [4] Idarucizumab is safe with only minor side effects, such as headaches, back pain, skin irritation and hypersensitivity. [5] Higher rates of thromboembolic events after idarucizumab use, once a concern, are now considered low, and similar to rates reported after major surgical procedures or hospitalization for uncontrolled bleeding. These thromboembolic events rates are similar to those of other reversal strategies. and due to delayed or non-resumption of anticoagulation. [6] Major limitations of idarucizumab use are availability in certain countries and its high cost. For example, the cost of a typical dose of 5 g (2 vials of 2.5 g) is ~ US$ 4400. We present here a case that challenges the conservative indications for idarucizumab and suggests its potential use to prevent major bleeding caused by dabigatran accumulation in AKI.

## Case report

**Fig. 1:**
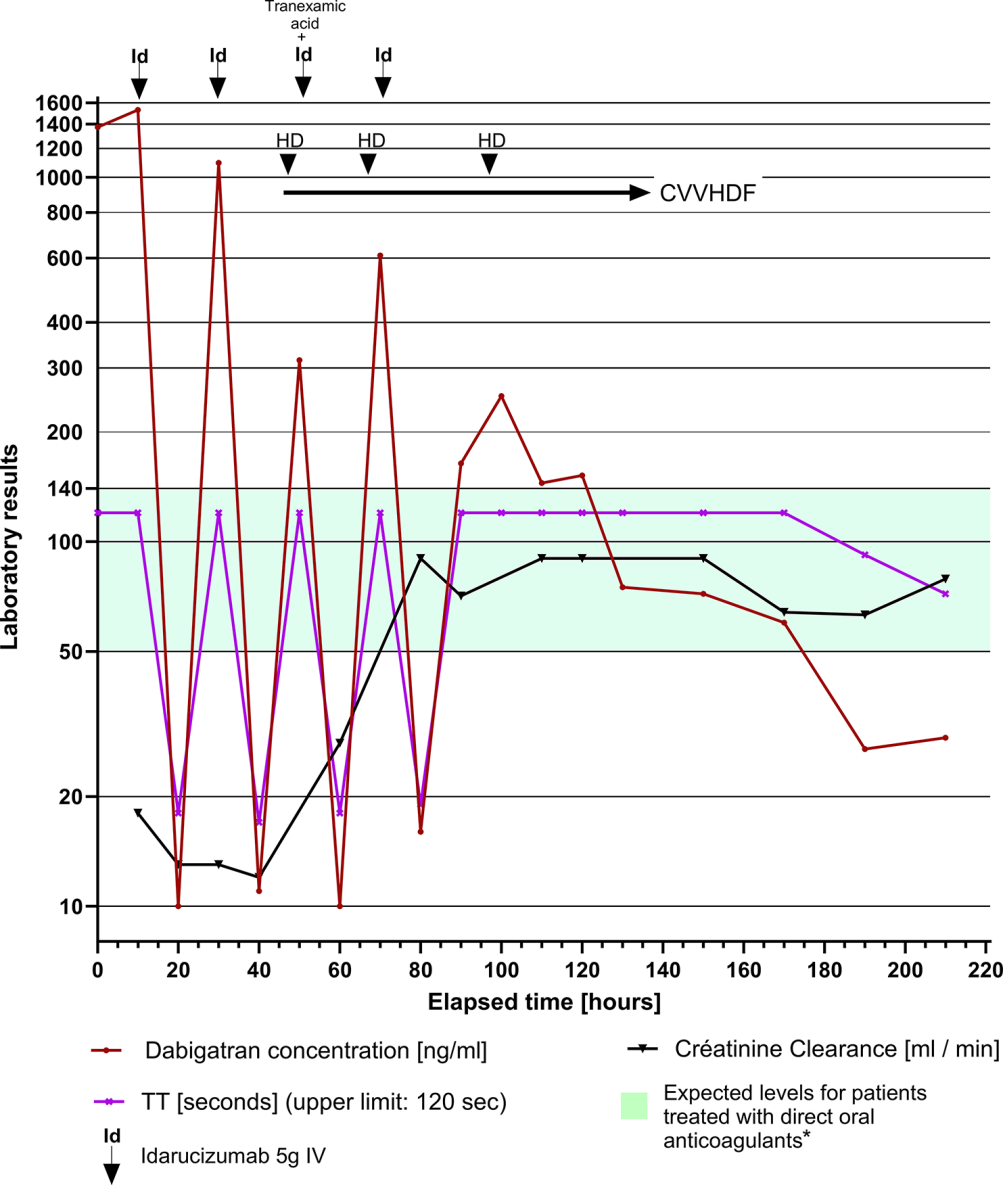
**Evolution of laboratory parameters over time**. Clinically relevant time points: H0: emergency department admission; H + 36 h: admission to continuing care unit with Glasgow Coma Scale 10/15; H + 44 h: major venous catheter bleeding and further Glasgow Coma Scale worsening 6/15; H + 50 h: subarachnoid hemorrhage with ventricular hemorrhage; H + 91 h: upper gastrointestinal bleeding; H + 94 h: Glasgow Coma Scale 15/15 HD, intermittent hemodialysis; CVVHDF, continuous venovenous hemodiafiltration; IV, intravenous; PT, prothrombin time; aPTT, activated partial thromboplastin time; TT, thrombin time. *: Moner-Banet T, Alberio L, Bart PA. Does One Dose Really Fit All? On the Monitoring of Direct Oral Anticoagulants: A Review of the Literature. Hämostaseologie. 2020;40(2):184–200

A 71-year-old morbidly obese woman with a history of diabetes, microcrystalline arthritis and atrial fibrillation anticoagulated by dabigatran 150 mg twice daily, was admitted to the emergency department of our tertiary care university hospital. She presented with a main complaint of new-onset asthenia, muscle weakness and impaired mobilization. She had no history of chronic kidney disease (baseline creatinine 63 µmol/L; N:44–80). She reported taking non-steroidal anti-inflammatory drugs during the past 5 days for joint pain. The initial clinical assessment found multiple ecchymosis and minor bleeding from a palatal ulcer, but no major bleeding or hemodynamic instability. Laboratory investigations revealed a serum creatinine level of 230 µmol/L, white blood count 24 G/L, C-reactive protein 340 mg/L, thrombin time > 120 s, and a dabigatran level of 1,372 ng/ml (expected level for patients treated with DOACs: 50–140 ng/ml), measured with HEMOCLOT™ Thrombin Inhibitors kit (Hyphen Biomed, Neuville-sur-Oise, France) on the hemostasis analyzer Sysmex CN-6000, with a lower limit of detection of 10 ng/ml. (Fig. 1; Supplementary Table 1). Imaging studies, including a computed tomographic (CT) scan without contrast of the abdomen, thorax, and head, did not reveal any signs of infection or bleeding. She was empirically covered with intravenous amoxicillin-clavulanic 2.2 g twice daily after blood cultures had been obtained.

On day 1 of admission, idarucizumab was not immediately administered as the patient did not meet its current indications. Regarding the AKI, the indication for acute dialysis was not retained in the absence of strict criteria and considering the probable functional origin. 12 h post admission (T + 12 h), the patient developed significant active bleeding at the site of a venous catheter in the right upper limb, with a risk of compartment syndrome, and a fall in hemoglobin of 30 g/l, indicative of a major bleeding episode according to the International Society on Thrombosis and Hemostasis criteria [7] and an indication for reversal. She responded well to 5 g of idarucizumab; her coagulation tests normalized (T + 20 h), and the plasma dabigatran concentration fell to < 10 ng/ml(Fig. 1). However, there was a rebound of the dabigatran concentration to 1,096 ng/ml (T + 30 h). The patient became suddenly confused and developed a new-onset neck stiffness. A repeat cerebral CT-scan showed a subarachnoid hemorrhage, with ventricular hemorrhage in both occipital horns of the 3rd ventricle and in the interpeduncular cistern. A second dose of 5 g idarucizumab was given at that time (T + 33 h) and she was subsequently transferred to the intensive care unit. A lumbar puncture confirmed a *Streptococcus agalactiae* meningitis.

After an initial normalization of her coagulation tests following the second dose of 5 g idarucizumab with a dabigatran concentration of 11 ng/ml, a second rebound was observed with an increased dabigatran concentration to 315 ng/ml, leading to the administration of a third dose of idarucizumab (T + 49 h) (Fig. 1). A third rebound to a dabigatran concentration of 610 ng/ml occurred (T + 68 h) and a fourth dose of idarucizumab was given In total, the patient received 20 g of idarucizumab. Repeated rebound effects following idarucizumab led to the initiation of continuous venovenous hemodialysis (CVVHD) (T + 48 h to T + 120 h) with the use of locoregional citrate anticoagulation and 3 intermittent hemodialysis (IHD) sessions to clear dabigatran (Fig. 1). The patient’s dabigatran levels remained low, without rebound effect 9 days after admission, 8 days after her first dose of idarucizumab. Her outcome was favorable, and her alertness and condition rapidly improved with idarucizumab, dialysis and antibiotic administration. She was discharged home 2 weeks after admission.

## Discussion

We present the case of a patient with a high risk of severe bleeding as bacterial meningitis is associated with a 2–3% intracranial bleeding risk overall, but as high as 25% in anticoagulated patients. [8] Anticoagulation is associated with a much greater risk of poor outcome or death, but earlier reversal is associated with greater survival. [9]. Some authors even argue for its preemptive reversal. [8, 10] The risk of a bleeding diathesis is further increased in anticoagulated patients with AKI. Due to its renal excretion, dabigatran’s half-life increases from 12–17 h in healthy individuals to 13–23 h in patients with moderate renal impairment (creatinine clearance [CrCl] 30–50 mL/min) and up to 22–35 h with severe renal insufficiency (CrCl < 30 mL/min) (Supplemental Fig. 1). [11] In individuals with a CrCl < 30 ml/min receiving a single dose of dabigatran, circulating levels remained detectable up to 96 h after drug administration, almost 4 times longer than healthy individuals. [3] In cases of massive accumulation, dabigatran levels remain above the expected levels for days (Supplemental Fig. 1), thus exposing patients to a prolonged increased bleeding risk. In such cases, the benefits of early anticoagulation reversal are expected to markedly reduce the risk of bleeding, even if not supported by current recommendations.

If reversal is the chosen strategy, the next question is to determine the best choice among the available options. Idarucizumab is administered as two consecutive 2.5 g intravenous boluses or perfusions over 5–10 min. However, a rebound elevation of dabigatran > 20 ng/ml, typically within the first 24 h, has been observed in up to 23% of patients. [5] This effect may be due to a free shift of dabigatran from the extravascular to the intravascular compartment, driven by a concentration gradient after elimination or saturation by idarucizumab in the intravascular compartment. Clotting parameters, in particular thrombin time, are consequently typically monitored closely during at least 24 h post-idarucizumab administration to detect this rebound. An initial concentration of dabigatran > 200 ng/ml has been recently linked to higher rates of rebound and hemostatic ineffectiveness in cases of bleeding after idarucizumab administration. [12] A modeling analysis showed that the administration of 2.5 g of idarucizumab as a 2-hour infusion markedly reduced the rebound effect compared with a bolus injection administration of 2.5 g, [13] constituting a potential new mode of administration if a sustained rather than an immediate reversal is needed. However, to our knowledge, no study has examined real-life differences between a bolus versus a 2-hour infusion of idarucizumab.

**Fig. 2:**
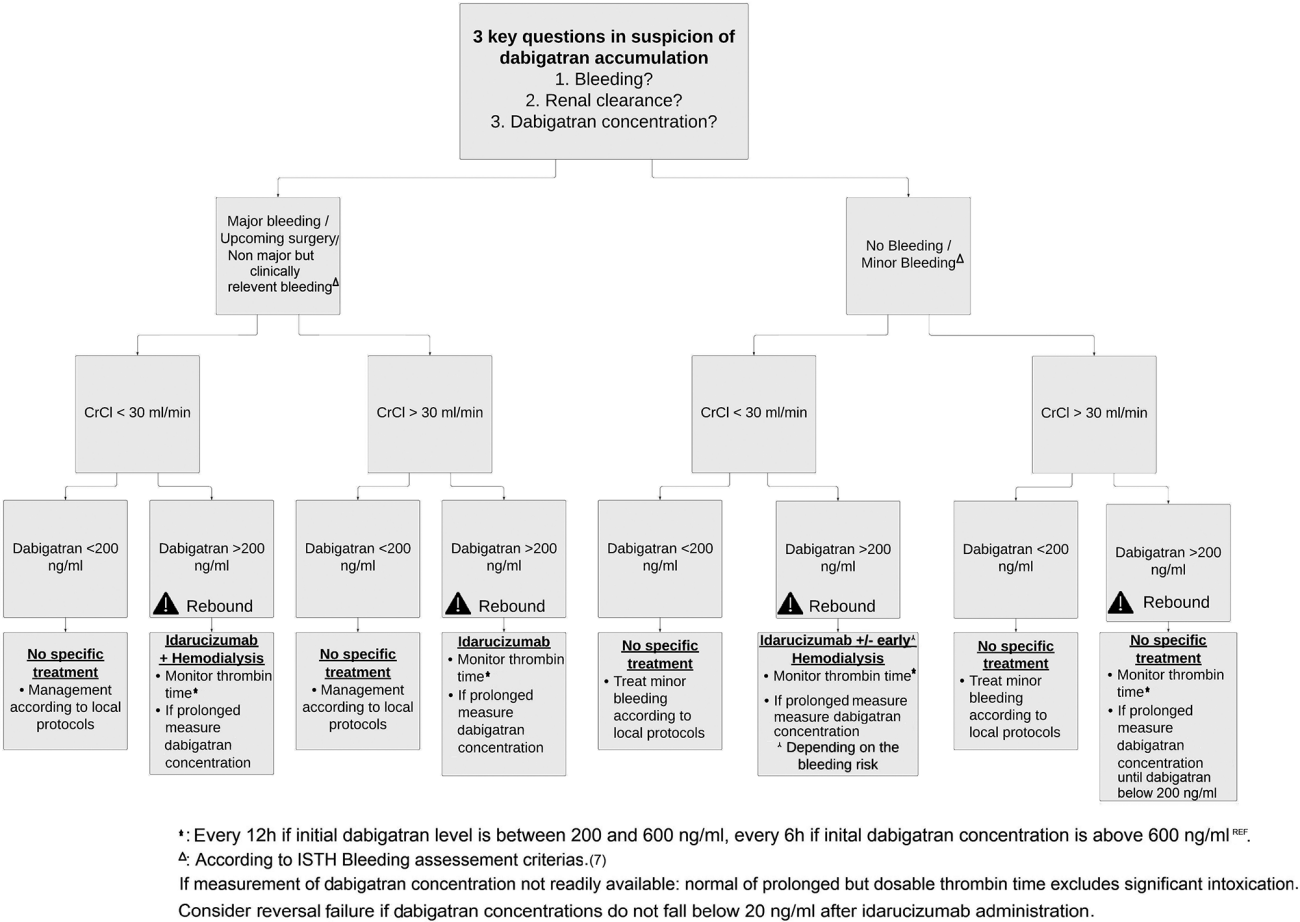
Proposed algorithm for the management of dabigatran overload, based in part on the latest publication of the French Society of Thrombosis and Haemostasis (Gendron et al., Usefulness of initial plasma dabigatran concentration to predict rebound after reversal. Haematologica. 2018 ;103(5):e226-9 and the latest French guidelines addressing DOAC reversal in emergency settings (https://sfar.org/gestion-de-lanticoagulation-dans-un-contexte-durgence/). Risk factors for dabigatran accumulation: (1) acute or acute-on-chronic renal failure; (2) interaction with glycoprotein P inhibitors (quinidine, dronedarone, ritonavir, tipranavir, nelfinavir, saquinavir, ciclosporin, tacrolimus, as well as ketoconazole or itraconazole); (3) Delirium / erroneous medication intake or administration

The 200 ng/ml threshold is largely exceeded in most published case reports of dabigatran accumulation and AKI. [14] In our opinion, a combination of IHD added to idarucizumab to clear dabigatran more rapidly and prevent a rebound effect and further bleeding complications should be proposed in patients with high dabigatran concentrations (Fig. 2), although evidence in the literature is sparce. A pharmacokinetic modeling analysis demonstrated that idarucizumab coupled with hemodialysis led to lower rates of dabigatran rebound. [13] Furthermore, when hemodialysis was started after a 2.5 g infusion of idarucizumab, the rebound was further reduced and anticoagulation was completely reversed. Measuring the initial concentration of dabigatran may provide clinicians with an approximate estimate of their patient’s duration of exposure concentrations above the expected levels and the associated increased hemorrhagic risk, thus guiding the reversal strategy. The choice of the type of extra-renal purification remains an open question. Some have advocated the use of sustained low-efficiency dialysis, [14] which consists in a prolonged IHD with a duration between 6 and 12 h and a reduced blood flow (100–150 ml/min), with the double advantage of being well supported in hemodynamically unstable patients, while better preventing rebounds when compared with IHD. [15]

This case clearly underscores the need for a reevaluation of idarucizumab indications and mode of administration to avoid rebound, including the place of early hemodialysis in treating dabigatran accumulation, particularly in non-surgical patients at high risk of bleeding. To our knowledge, there are no studies assessing the potential use of idarucizumab in preventing life-threatening bleeding in such patients, particularly those with a high initial concentration of dabigatran due to AKI. This gap in knowledge warrants further investigation to enhance patient care and safety when dabigatran accumulation poses a significant bleeding risk.

### Electronic supplementary material

Below is the link to the electronic supplementary material.

Supplementary Material 1

Supplementary Material 2

## Data Availability

No datasets were generated or analysed during the current study.
