# Supplementary figures and images for: Dabigatran accumulation in acute kidney injury: is more better than less to prevent bleeding? A case report
Source: Int J Emerg Med. 2024 Jul 17;17:91. doi: 10.1186/s12245-024-00677-3 (PMC11253475; doi:10.1186/s12245-024-00677-3)

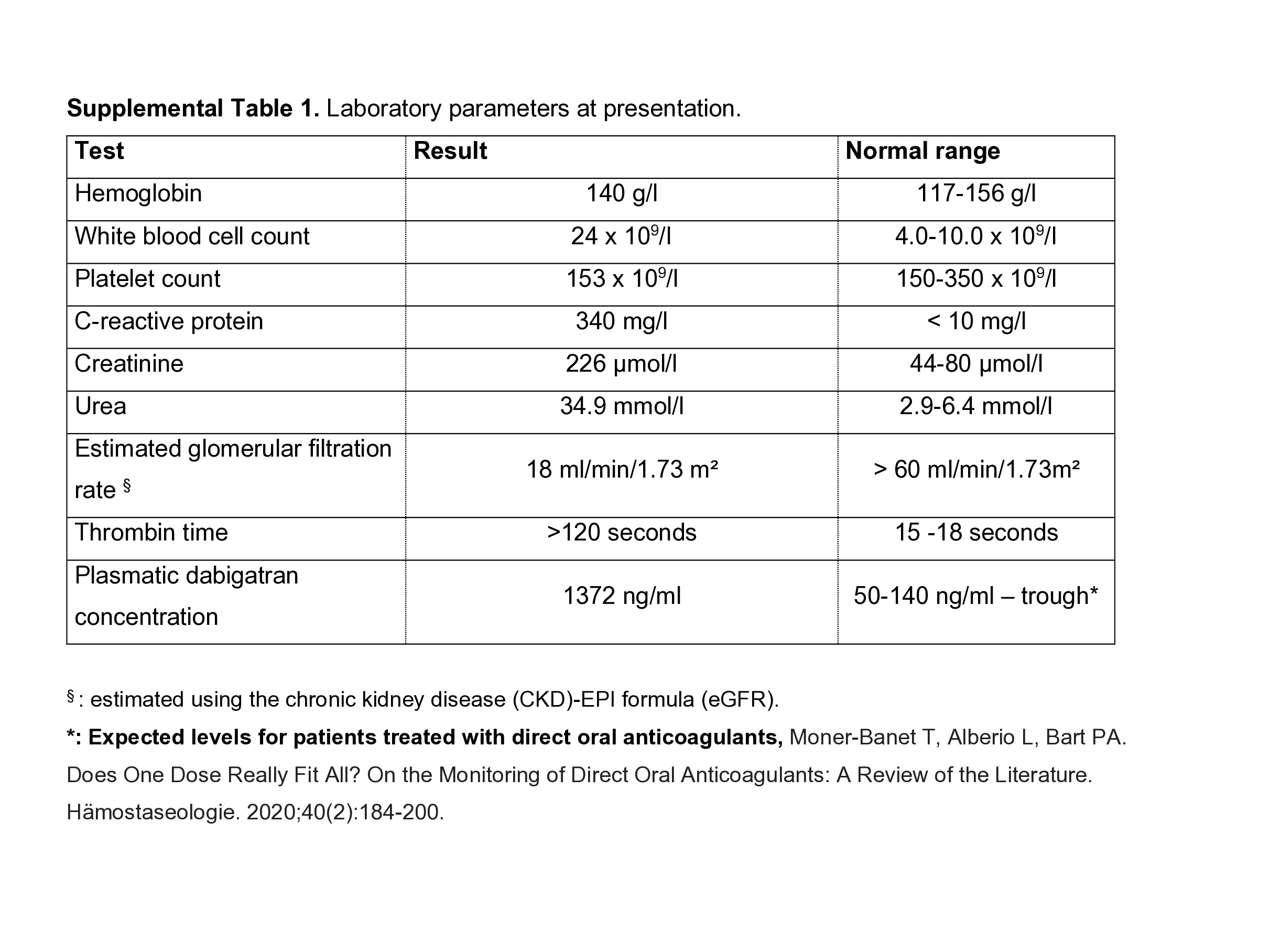

Supplement: Supplementary file 2 — Supplementary Material 2 [file 12245_2024_677_MOESM2_ESM.jpeg]
